# Never Events in UK General Practice: A Survey of the Views of General Practitioners on Their Frequency and Acceptability as a Safety Improvement Approach

**DOI:** 10.1097/PTS.0000000000000380

**Published:** 2017-04-27

**Authors:** Susan J. Stocks, Rahul Alam, Paul Bowie, Stephen Campbell, Carl de Wet, Aneez Esmail, Sudeh Cheraghi-Sohi

**Affiliations:** From the ∗ NIHR Greater Manchester Primary Care Patient Safety Translational Research Centre, University of Manchester, Manchester; † Centre for Primary Care, Institute of Population Health, University of Manchester, Williamson Building, Manchester; ‡ NHS Education for Scotland, Glasgow; § Institute of Health and Wellbeing, University of Glasgow, Glasgow, United Kingdom; ∥ Centre for Research and Action in Public Health (CeRAPH), University of Canberra, Bruce, Australian Capital Territory; ¶ School of Medicine, Gold Coast Campus, Griffith University, Nathan, Queensland, Australia.

**Keywords:** never events, general practice, primary care, patient safety, clinical risk

## Abstract

**Background:**

Never events (NEs) are serious preventable patient safety incidents and are a component of formal quality and safety improvement (Q&SI) policies in the United Kingdom and elsewhere. A preliminary list of NEs for UK general practice has been developed, but the frequency of these events, or their acceptability to general practitioner (GPs) as a Q&SI approach, is currently unknown. The study aims to estimate (1) the frequency of 10 NEs occurring within GPs' own practices and (2) the extent to which the NE approach is perceived as acceptable for use.

**Methods:**

General practitioners were surveyed, and mixed-effects logistic regression models examined the relationship between GP opinions of NE, estimates of NE frequency, and the characteristics of the GPs and their practices.

**Results:**

Responses from 556 GPs in 412 practices were analyzed. Most participants (70%–88%, depending on the NE) agreed that the described incident should be designated as a NE. Three NEs were estimated to have occurred in less than 4% of practices in the last year; however, two NEs were estimated to have occurred in 45% to 61% of the practices. General practitioners reporting that a NE had occurred in their practice in the last year were significantly less likely to agree with the designation as a NE compared with GPs not reporting a NE (odds ratio, 0.42; 95% CI = 0.36–0.49).

**Conclusions:**

The NE approach may have Q&SI potential for general practice, but further work to adapt the concept and content is required.

## Abstract 2

Supplemental digital content is available in the text.

A substantial minority of patients unintentionally experience harm as a result of their interactions with health care systems, including general practice.^1^ Patient safety incidents in general practice are thought to be a relatively frequent occurrence, but most do not result in significant harm.^2^ Many patient safety incidents may be preventable, creating a powerful rationale for initiatives to improve patient safety.^3^ The World Health Organisation Safer Primary Care Expert Working Group recently recommended a systems approach toward reducing the occurrence of patient safety incidents.^4,5^

Improving patient safety in general practice may require adapting approaches from safety-critical industries and other health care settings. There are many practical examples where this has been achieved, including (1) the development and testing of a number of validated instruments to measure perceptions of safety culture,^6–8^ (2) application of the “care bundle” approach to improve chronic disease management,^9^ and (3) application of the trigger review method to patients' medical records.^10^

A further example of a quality and safety improvement (Q&SI) initiative is the introduction of a ‘Never Events’ policy in 2009 to secondary care provided by the UK National Health Service (NHS).^11,12^ A never event (NE) is a serious, largely preventable patient safety incident that should not occur if the available preventative measures were implemented by health care organizations.^13^ They are relatively rare with 308 formal reports of NE occurring during 2014/2015 in England.^14^ Despite their apparent rarity, NE policies are considered worthwhile because of their potential benefits in terms of (1) increasing awareness of priority patient safety issues, (2) providing organizational support to proactively implement preventative measures, and (3) formally acknowledging and dealing with serious patient safety incidents. Never events could help build a positive safety culture in general practice at the practice level by prioritizing incidents for significant event analysis (SEA)^15^ and/or be used to proactively review local safety systems. Formal reporting of NE could inform policy makers, researchers, educators, and frontline teams about the nature, scale, and scope of patient safety incidents.

Ten NE specifically for general practice have been developed (Box 1,^16^) but the frequency of these proposed NE is currently unknown. Given that approximately 90% of patient interaction is with NHS primary care services, it seems very likely that serious, preventable patient safety incidents do occur and some measure of the frequency would be desirable to inform patient safety initiatives in general practice.

Box 1. List of NE labels and description^16^

1. Prescribing aspirin for a patient 12 years or younger

- Prescribing aspirin for a patient 12 years or younger (unless recommended by a specialist for specific clinical conditions, e.g., Kawasaki disease)

1. Methotrexate prescribed daily rather than weekly

- Prescribing Methotrexate daily rather than weekly (unless initiated by a specialist for a specific clinical condition, e.g., leukemia)

1. Adrenaline is NOT available when needed

- Adrenaline/epinephrine is NOT available within minutes when clinically indicated for a medical emergency in the practice or GP home visit

1. Prescribed teratogenic drug when pregnant

- Prescribing a teratogenic drug to a patient the clinician knows to be pregnant (unless advised to do so by a clinical specialist)

1. Prescribed HRT and has intact uterus

- Prescribing systemic oestrogen-only hormone replacement therapy for a patient with an intact uterus

1. Cancer referral not sent

- A planned referral of a patient, prompted by clinical suspicion of cancer, is not sent

1. Ambulance transport is not arranged

- Ambulance transport is not arranged if this had been agreed when deciding to admit a patient as an emergency

1. Needle stick injury due to sharps disposal failure

- A needle stick injury due to a failure to dispose of “sharps” in compliance with national guidance and regulations

1. Prescribing when adverse reaction recorded

- Prescribing a drug to a patient that has correctly been recorded in the practice system as having previously caused her/him a severe adverse reaction

1. Abnormal investigation result is not reviewed

- An abnormal investigation result is received by a practice but is not reviewed by a clinician

This study aims to (1) to determine the annual frequency of the proposed NE (Box 1) as estimated by UK general practitioners (GPs), (2) to explore the extent to which the NE approach is acceptable to GPs, (3) to examine the relationship between GP's opinions and estimates as described in aims 1 and 2 and the characteristics of the GPs and their practices.

## METHODS

### Questionnaire Development and Testing

The survey was developed in an iterative manner. For each NE (Box 1), the GPs were asked Q1 to Q5 (Box 2) with the option to provide free text comments. Briefly, the questionnaire focused on respondents' previous experiences of NE, their frequency of occurrence, estimates of risk of reoccurrence, perceptions of each incident as a NE, and actions taken in response to a NE. General practitioners were also asked to provide information about their practices and themselves. An initial version was piloted in Scotland by 15 GPs in December 2013; the final version survey was not substantially altered after the pilot.

Box 2. Questions asked for each never event (Q1–Q4) and for all never events (Q5)

Q1. Based on your experience, please indicate the number of times this event has occurred in your current practice in the last year?

Q2. Based on your experience, please indicate the number of times this event has occurred in your current practice or any other practice you have ever worked?

Q3. Do you think this incident is a “never event”?

Q4. Please estimate the risk of this event occurring in your practice in the next 5 years

Q5. If you become aware in the future that any of the previous incidents have occurred in your practice, which of the following actions would you be prepared to undertake?

- Informal discussion with colleague(s) involved in the incident
- A significant event analysis
- Submit a formal incident report to a local health authority or other organization
- Discuss the incident at a practice meeting
- Adopt a “watch-and-see” approach if no patient harm occurred

### Setting and Participants

The questionnaire was administered concurrently to 519 general practices in Greater Manchester and all GP educational supervisors in Scotland (709 GPs within 332 practices representing approximately one quarter of all Scottish GPs) between April 1, 2014, and May 31, 2014.

### Data Collection

The questionnaire was completed online in Scotland, and nonresponders were followed-up on 2 occasions at 7-day intervals. In Greater Manchester (GM), the questionnaire was distributed by post and nonresponders were followed up on 2 occasions at 2-week intervals.

### Statistical Methods

**FIGURE 1:**
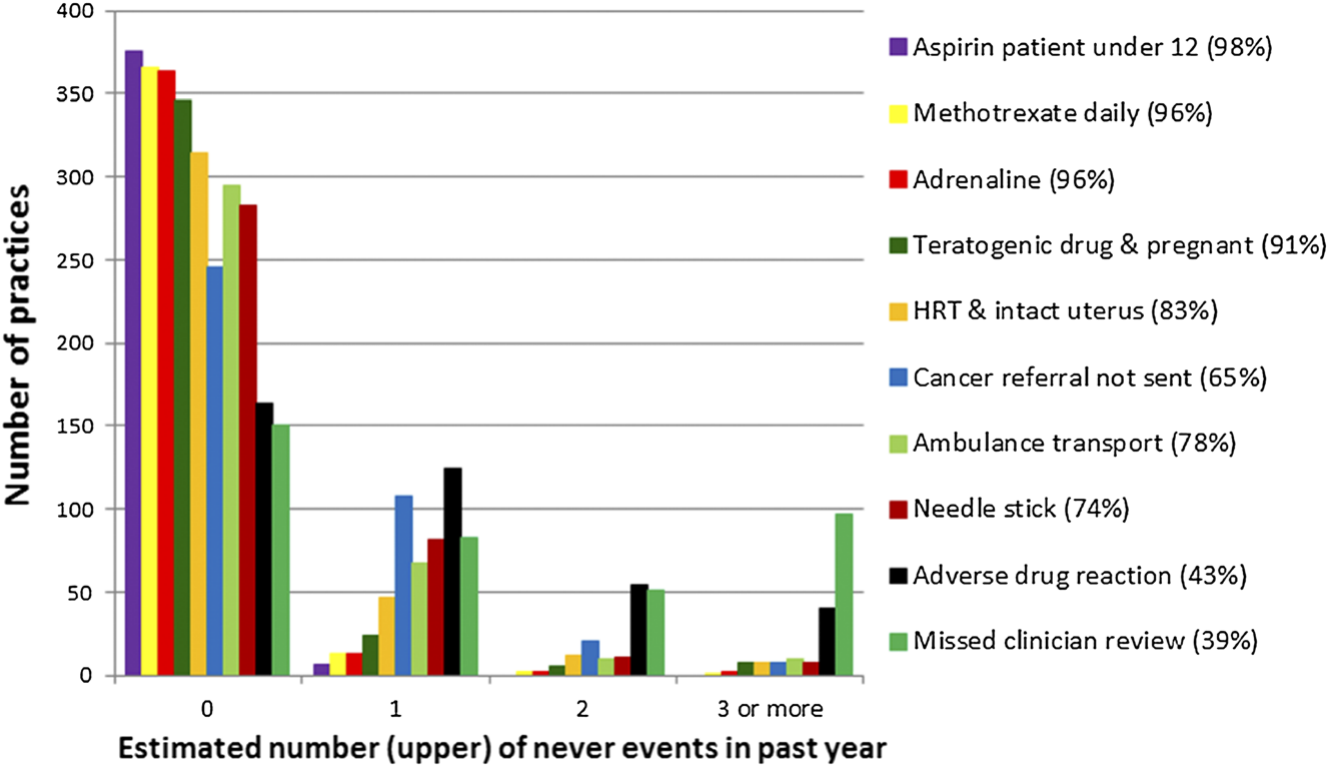
Frequency of NEs occurring in the past 12 months as estimated by GPs (% practices with 0 NE). The higher estimate is shown in cases of disagreement between GPs within the same practice.

The proportion of GPs or practices reporting NE to have occurred was reported. Estimates were collapsed into either categorical outcomes (frequency of NE = 0, 1, 2, 3, or more, Fig. 1) or binary outcomes (NE did not occur or occurred at least once, Table 1). When asking about the frequency of NE occurrence during the last year (Q1, Box 2), only GPs that had worked for at least 1 year in their current practice were included (Table 1). For the practice estimates where there were disagreements in estimated frequencies between GPs, the most frequently occurring estimate was used. If there were equal numbers of contradictory estimates of NE per practice (i.e., practices where 2 or 4 GPs had completed the questionnaire), both the lowest and highest estimates were reported (Table 1). To adjust the GP estimates for each NE for practice location (GM or Scotland) and allow for the clustering of GPs within practices, a two-level logistic regression model was used (Tables 1, 2). The practice location was included as a fixed effect and therefore adjusted the reported estimates, whereas the practice identifier was included as a random effect and thereby provided more accurate confidence intervals (Tables 1, 2). For the question asking about the level of agreement with the designation of the event as a NE (Q3, Box 2), the responses were dichotomized into a group expressing some level of agreement (“yes,” “probably,” and “possibly”) and a group expressing definite disagreement (“no,” Table 2). The odds ratio that a GP would agree with the designation as a NE relative to NE1 (prescribing aspirin for a patient ≤12 years, Box 1) was estimated using a standard 1-level logistic regression model (Table 2).

**TABLE 1:**
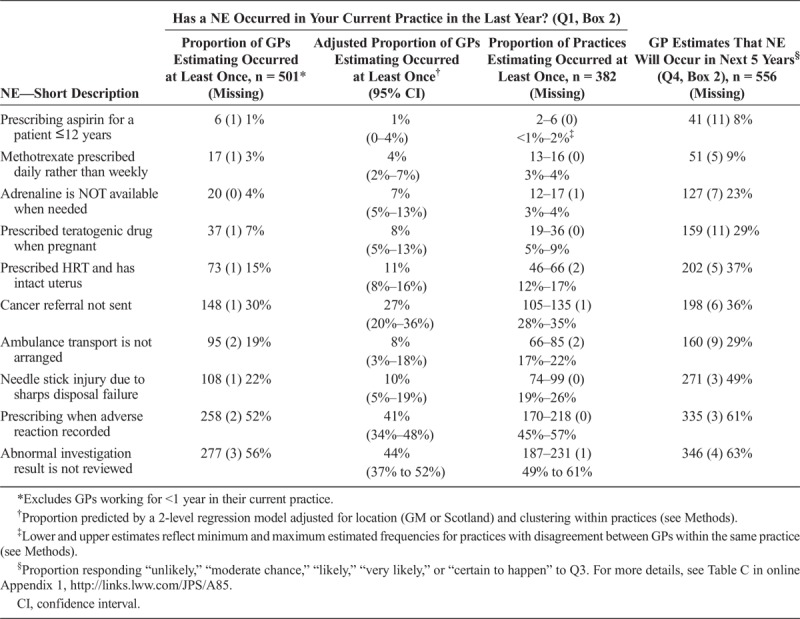
General Practitioner Estimates of the Frequency of NEs Occurring in the Last Year and in Future

**TABLE 2:**
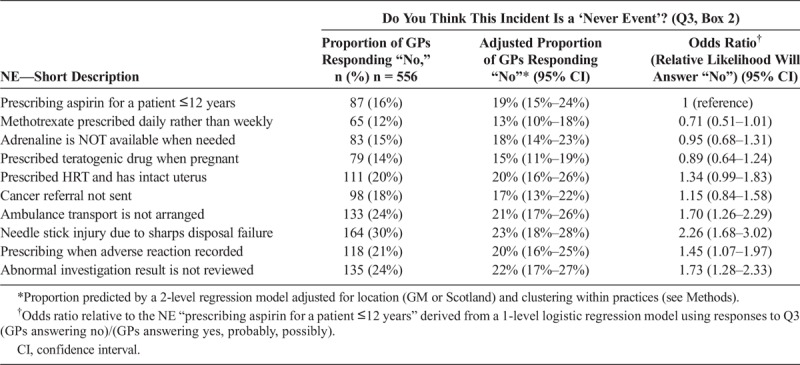
The Number of GPs Disagreeing With the Designation as a NE (Answered “No” to Q3, Box 2)

To examine the relationship between the dichotomized responses to Q1 to Q4 and the characteristics of the GPs and their practices across all the NE, 4 versions of a three-level logistic regression model were fitted (Table 3). The three-level model allows for different outcomes for each NE and takes clustering of GPs within practices in to account by including categorical variables identifying each NE and practice as random effects in the model.

**TABLE 3:**
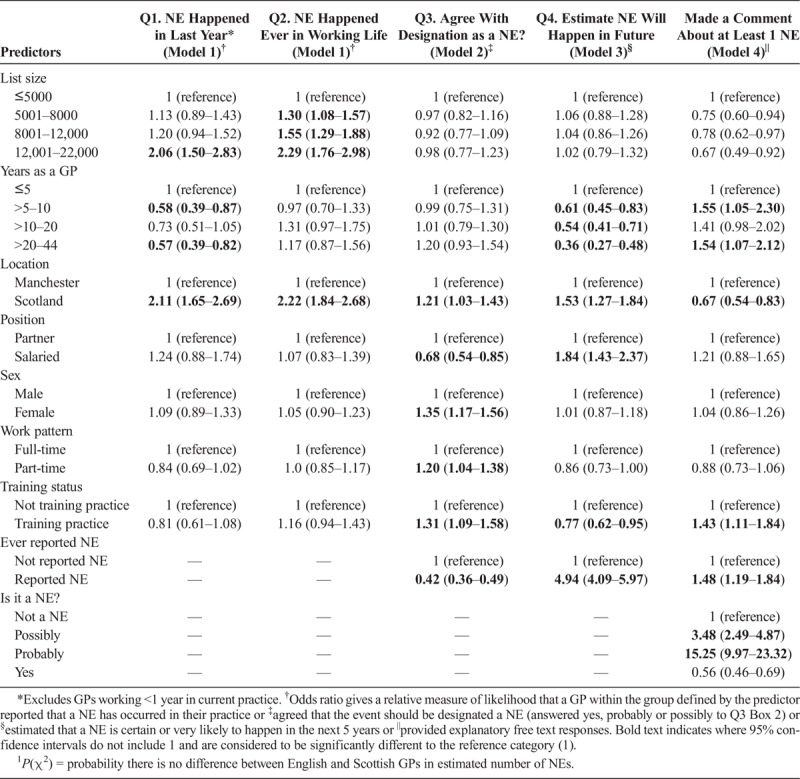
Adjusted Odds Ratios for GP and Practice Level Predictors From a 3-Level Logistic Regression Model With Random Effects at the NE and Practice Level

Model 1—associations between reporting a NE (Q1&2, Box 2) and GP/practice characteristics

Model 1 examined the associations between a binary outcome variable indicating whether or not a NE occurred at least once or did not occur and the following predictors: years worked as a GP, partner or salaried GP, sex, part or full-time working pattern, practice list size, location (Scotland or GM), and whether the practice was an accredited training practice (all Scottish practices were training practices). The odds ratios from the regression model give a relative measure of how likely GPs were to estimate that a NE had occurred in their practice according to the characteristics listed previously.

Model 2—associations between agreement with the designation as a NE (Q3, Box 2), the reporting of a NE in past year (Q1, Box 2), and GP/practice characteristics

Model 2 examined the associations between a binary outcome variable indicating whether or not a GP agreed with the designation of the event as a NE (answered “yes,” “probably,” and “possibly” to Q3) or disagreed (answered “no” to Q3). The predictors were the same as in model 1 (listed previously) plus a variable indicating whether or not the GP had estimated a NE to have occurred in the last year. The odds ratio for this additional variable estimates the impact of working in a practice where a NE had occurred in the last year on the GP's opinion about agreement or disagreement with the designation of the event as a NE (Q3, Table 2).

Model 3—associations between estimation that NE will occur within the next 5 years (Q4, Box 2), the reporting of a NE in past year (Q1, Box 2), and GP/practice characteristics

Model 3 examined the associations between a binary outcome variable indicating GP's opinion about the likelihood that a NE would occur in the next 5 years. The responses to Q4 were dichotomized into groups estimating that it was very unlikely that a NE would occur in the next 5 years (no chance or very unlikely to happen) versus those estimating that it could happen (unlikely through certain to happen). The predictors were the same as in model 2 (listed previously).

Model 4—associations between providing free text comments, the designation as a NE (Q3, Box 2), the reporting of a NE in past year (Q1, Box 2), and GP/practice characteristics

Model 4 examined associations between providing free text comments, or not, and used same predictors as model 2 (listed previously) plus a variable indicating the GPs level of agreement with the statement that this was a NE (Q3). The odds ratios for the predictors help describe the characteristics and opinions of the GPs who provided free text comments.

### Content Analysis

A bottom-up (inductive) approach was used to identify similar topics within the comments. One author (S.J.S.) read the comments in a random order, that is, not within their NE grouping, and identified the most frequent topics. Each comment was coded accordingly, and as new topics emerged, the comments were recorded and similar codes were merged. The process ended when each comment had at least 1 code although some comments had multiple codes when several topics were addressed in the same comment. The coding was checked by a different author (R.A.) and any disagreements were resolved by discussion.

## RESULTS

### Response Rates

In Scotland, 283 GPs representing 215 practices responded to the questionnaire (practice response rate = 65%, GP response rate = 40%). Two GPs were excluded from the analysis (1 retired and 1 locum GP). In GM, 282 GPs representing 202 practices responded (practice response rate = 39%; GP response rate not available as invitations were at the practice level). Seven GM physicians were excluded from the analysis (2 did not work in general practice, 3 were other types of clinician working in general practice, 1 locum GP, and 1 GP only completed 1 question). After these exclusions, questionnaires from 281 GPs in 214 practices in Scotland and 275 GPs in 198 practices in GM were analyzed (412 practices in total). More than 1 GP completed the questionnaire in 109 (26%) of 412 practices. In 96 (23%) of 412 practices, 2 GPs completed the questionnaire; in 12 (3%) of 412, 3 GPs; and in 1 (1%) of 412, 4 GPs.

### Professional Characteristics and Practice Demographics

All Scottish GPs were educational trainers; therefore, their practices were designated as training practices. In GM, 48% of practices were training practices (91/191, 7 not known). Across both locations, most GPs were partners rather than salaried (90%; 499/555, 1 missing), 56% were working part-time (312/555, 1 missing), and there were equal numbers of each sex (51% male 284/554, 2 missing). General practitioners tended to be experienced, particularly the Scottish GPs (mean [SD] time worked as a GP; Scotland =19.1 [7.4] years, GM =17.2 [9.4] years, *P* < 0.001). There were no differences in the mean list size between Scotland (7482; [2997]) and GM (7481 [3848], *P* = 0.997), but Scottish practices had more partners (5.3 [2.0]) compared with GM (3.9 [2.0], *P* < 0.001).

### Estimated Frequency of NE

The estimated frequency of occurrence for each NE during the last year is shown in Figure 1 (upper estimates) and Table 1. Four practices were excluded because the sole GP responding had been in post for less than 1 year. Furthermore, due to an administrative error, 45 (23%) of the 198 GM practices and 48 (17%) of 275 GM GPs were not asked to estimate the frequency of NEs during the last year (Q1) but the full response set was available for all other questions.

The proportion of practices estimating that a NE had occurred in the past year ranged from less than 1% to 61% (comparing lower estimate for prescribing aspirin for a patient <12 years with upper estimate for abnormal investigation result not reviewed, Table 1). Three of the 10 NEs were estimated to have occurred in the last year in 4% or fewer practices (“prescribing aspirin for a patient <12 years,” “methotrexate daily rather than weekly,” “adrenaline is not available when needed,” Table 1). Conversely, 2 NEs were estimated to have occurred in the last year in 45% to 61% of practices (“abnormal investigation result not reviewed,” “prescribing when adverse reaction recorded,” Table 1).

### Estimated Likelihood That NE Will Occur in Future, Level of Agreement With the Designation as **a NE**, and Actions After **a NE**

The proportion of GPs who estimated that NEs were definitely or likely to happen in the next 5 years ranged from 8% (prescribing aspirin for a patient <12 years) to 63% (abnormal investigation result not reviewed, Table 1). A more detailed breakdown of the response to this question is shown in Table C, online appendix 1, http://links.lww.com/JPS/A85. The proportion of GPs responding “no” to Q3 (Do you think this incident is a ‘never event’?) ranged from 12% to 30% (Table 2). Of the 556 GPs, 551 (99%) reported that they would undertake a SEA in response to a NE and 141 (25%) would also submit a formal incident report. Of the 5 GPs remaining, 2 (<1%) would discuss the NE at a practice meeting and 3 (<1%) did not report any actions, but other GPs in the same practice reported that they would take one of the actions listed previously.

### Results of the Multilevel Regression Models

The relative importance of the patient and practice characteristics (predictors) in determining the outcome (response to Q1–4, etc) are shown as odds ratios generated by the regression models in Table 3. If the 95% confidence intervals for an odds ratio do not overlap the reference category (i.e., 1), it is considered to be significantly different to the reference category (and highlighted in bold). The *P* value for an odds ratio significantly different to the reference category is therefore less than 0.05 (but could be smaller). The odds of estimating a NE had occurred in the last year (Q1) were doubled in practices with a list size of more than 12,000 relative to those less than 5000. Scottish GPs and less experienced GPs (less than 5-year work experience) were significantly more likely to report a NE (model 1, Table 3).

General practitioners who had estimated that a NE had occurred in the last year were significantly less likely to agree that it should be designated as a NE but female GPs, part-time GPs, Scottish GPs, partners rather than salaried GPs and GPs working in training practices were all significantly more likely to agree that the event should be described as a NE (model 2, Table 3).

The strongest predictor of a GP being of the opinion that a NE would occur in the next 5 years (Q4) was the frequency of past occurrences. Less experienced, salaried, and Scottish GPs were significantly more likely to estimate that a NE would occur in the next 5 years (model 3, Table 3).

General practitioners who were undecided about the designation as a NE were most likely to provide free text comments (GPs answering “probably” to the Q4 is it a NE were 15 times more likely to provide a comment than those answering “no” and 30 times more likely than those answering “yes”). General practitioners who answered that a NE had occurred in their practice, GPs working in a training practice and more experienced GPs were more likely to comment, and Scottish GPs were less likely to comment than GM GPs (model 4, Table 3).

### Results of Content Analysis of Free Text Comments

In total, 1025 comments were provided, most made 1 main point (72%) but some addressed multiple points (28%). The comments tended to be very specific to an individual NE, and altogether, they fell into 28 groups making a substantively similar point (Table 4). Some comments suggested changes to the NE to make it more acceptable (Table A, online Appendix 1, http://links.lww.com/JPS/A85). A detailed summary of the categories of comments for each NE is shown in Tables B1 to B10 (online Appendix 1, http://links.lww.com/JPS/A85).

**TABLE 4:**
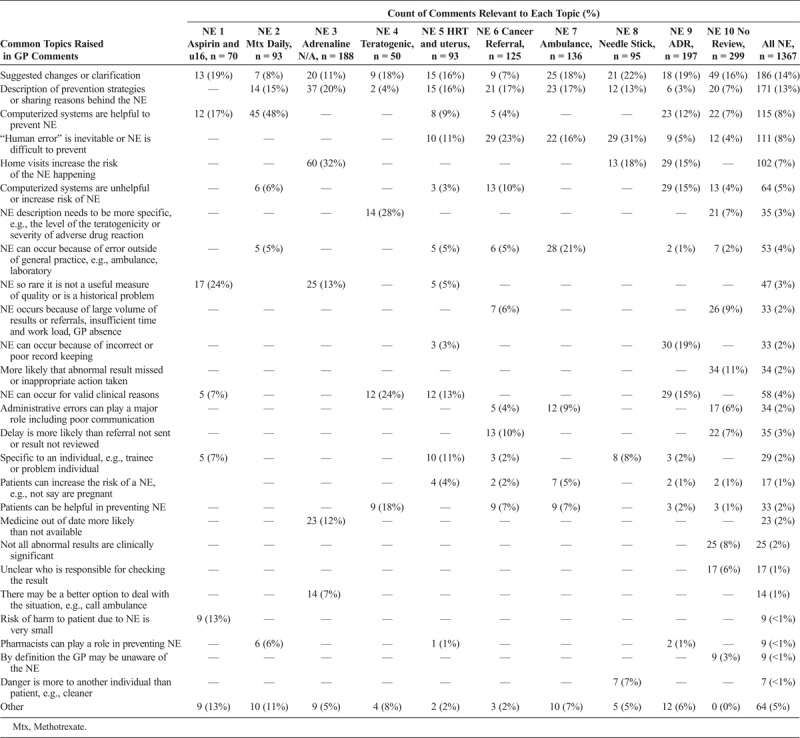
Counts of Themes Identified Within the Comments Provided by GPs

## DISCUSSION

### Summary of Results

The study findings suggest that the designation of a NE as a NE is dependent on the individual/type of NE and that on the whole, NEs were reportedly rare. Although GPs were more likely to disagree with the NE label for the more frequently occurring NEs, this was not in proportion to their increased frequency of occurrence. For example, a “cancer referral not sent” was approximately 10 times more likely to have occurred than “methotrexate prescribed daily rather than weekly” (Table 1), but GPs were only approximately 1.5 times more likely to disagree with the designation as a NE (Table 2). Most GPs, however, remained unconvinced that the risk can be eliminated for any of the NEs (Table 1, Table C in online Appendix 1, http://links.lww.com/JPS/A85). General practitioners do, however, seem to take the actual and potential occurrence of such events seriously given that 99% stated an intention to undertake a SEA^17^ after a NE. Free text comments originated mainly from GPs who were undecided about the labeling of the event as a NE. Opinions varied widely with some GPs commenting that the risk of serious harm was extremely low, whereas other GPs suggested that the NE should be more stringent. Some GPs felt that the NE description was placing a burden of responsibility on them that was not intended by the description of the NE, for example, that they should be responsible for the actions of a laboratory or the ambulance service. There were differences in opinion about the level of responsibility a GP should take for the actions of nonmedical staff.

### Strengths, Limitations, and Generalizability of the Study

This is the first study to attempt to estimate the frequency of a preliminary set of NEs for UK general practice and as such will help inform whether or not a NE policy should be rolled out across UK general practice. Despite its potential contribution, the study does have some limitations. Firstly, unfortunately, some GM GPs were not asked the first question because of a mistake when printing the questionnaires and these practices were excluded from the analysis of this question. Secondly, included practices may not be generalizable to all of the UK general practice; participating practices tended to be larger than their respective national averages (list size 7482 versus 5622 in Scotland^18^ and 7481 versus 6487 in England^19^). Furthermore, the sample is self-selected and includes only GP trainers in Scotland. In GM, training practices were more likely to respond to the survey (48% compared with approximately 40% of all practices in Central and South Manchester). This might be a source of bias given that training practices were more likely to agree with the description as a NE, were more optimistic about the likelihood of NE happening in future, and were more likely to make a free text comment (Table 3). Thirdly, we must emphasize that these results and analyses are based on retrospective and subjective estimates by GPs, and we do not know how often NEs might truly occur in primary care. Furthermore, certain events may more easily be recalled than others and may also bias the reported frequencies. The estimates are intended to inform the reader about the potential usefulness of the NE approach in demonstrating that GPs recognize these events and state that they do happen, rather than estimate the actual frequency of occurrence.

### Implications for Research and Practice

Our results show that the NE approach does not transfer easily from secondary care to primary care. Nonetheless, NEs may have a role in general practice with some refinement of the content and purpose. For example, NEs 1 to 5 (Box 1) are predominantly errors of commission; they rarely occur and GPs are more likely to agree with their designation as a NE. They are too rare to be used to identify practices in need of intervention but could be used at a system level to draw attention to patient safety. Focusing on rare events at the practice level may not be the best use of limited resources; indeed, it seems counterintuitive.^19^ The more frequently reported NEs (6–10, Box 1) are predominantly errors of omission and GPs are less likely to agree with their designation as a NE. In general practice, however, mild to moderate harm is more commonly associated with errors of commission and severe harm is more commonly associated with errors of omission such as misdiagnosis or delayed diagnosis.^5^ This contrasts with secondary care where NEs are predominantly errors of commission. Thus, although NEs (6–10) are more likely to be associated with more serious harm and occur more frequently, they are less likely to be recognized as NE by GPs. Never events 6 to 10 could be expanded into a set of markers for Q&SI, possibly by going back to the original 50 NEs from which this list of 10 was developed.^16^ Selected subsets could then be adapted locally to suit individual contexts and preferences, and weighting of particular NE might help make them acceptable to GPs. It is also difficult to compare the rates estimated here with those reported in secondary care as the denominators differ, for example, 340 million general practice consultations annually compared with 19 million episodes for patients admitted to hospital.^20,21^ A crude scaling up (assuming 8000 practices in England) implies approximately 50 occurrences of “prescribing aspirin for a patient ≤12 years” and 300 of “methotrexate prescribed daily rather than weekly” per year compared with approximately 300 NEs reported in English hospitals in 2014.^14^ Secondly, the potential to cause serious harm after a NE in general practice is less clear cut than in secondary care; there may the opportunity to remove or mitigate the consequences of the NE in the future, and all the definitions include caveats and exceptions.

In our study, the content analysis revealed that the locus of responsibility for the occurrence of NEs was also an issue. In secondary care, the lines of responsibility and boundaries of operations may be more easily defined as a closed system, whereas general practice is more diffuse and potentially viewed in comparison with secondary care, as an open system.^22^ Other work, for example, has pointed out the importance of receptionists in reducing the likelihood of a medical error.^23^

Finally, given that GPs are less likely to agree with the NE designation if they reported a NE in their practice, it seems possible that they might view the NE as a negative comment on their practice and would need to be reassured that the aim of a NE policy is to identify weaknesses in the system and prevent NEs from occurring, rather than a way to punitively judge general practice. Ostensibly, replacing the “NEs” label with one that better reflects the missed opportunities to improve patient safety might improve their acceptability to GPs.

## CONCLUSIONS

In a resource-limited and overstretched system, there may be a trade-off when addressing comprehensive patient safety. We suggest that further work could explore expanding the list of more frequently occurring NE (e.g., 6–10) as a Q&SI approach at a practice level and the rarer NE (1–5) could be useful for surveillance at a system level and to draw attention to broader safety issues. The “NEs” label could be replaced with one that better reflects the missed opportunities for NE 6 to 10, for example, “serious preventable events,” but could be retained for NE 1 to 5.

## Supplementary Material

SUPPLEMENTARY MATERIAL
