# Supplementary material for: Never Events in UK General Practice: A Survey of the Views of General Practitioners on Their Frequency and Acceptability as a Safety Improvement Approach
Source: J Patient Saf. 2017 Apr 27;15(4):334–42. doi: 10.1097/PTS.0000000000000380 (PMC5542029; doi:10.1097/PTS.0000000000000380)
Supplement: SUPPLEMENTARY MATERIAL [file pts-15-334.s001.docx]

**Never Events in UK general practice: a survey of the views of General Practitioners on their frequency and acceptability as a safety improvement approach**

Susan J Stocks, PhD^1^, Rahul Alam, PhD^1,2^, Paul Bowie, PhD^3,4^, Stephen Campbell, PhD^1,2,5^, Carl de Wet, MRCGP, MFPHM^6^, Aneez Esmail, PhD, MRCGP, MFPHM^1,2^, Sudeh Cheraghi-Sohi, PhD^1,2^

1. NIHR Greater Manchester Primary Care Patient Safety Translational Research Centre, University of Manchester, 7th Floor: Williamson Building, Manchester M13 9PL, UK

2. Centre for Primary Care: Institute of Population Health, University of Manchester, 7th Floor Williamson Building, Manchester M13 9PL, UK

3. Medical Directorate, NHS Education for Scotland, 2 Central Quay, Glasgow G3 8BW, UK.

4. Institute of Health and Wellbeing, University of Glasgow, Glasgow G12 0XH, UK

5. Centre for Research and Action in Public Health (CeRAPH), University of Canberra, Building 22, Floor B, University Drive, Bruce, ACT 2617, Australia

6. School of Medicine, Gold Coast Campus, Griffith University, Queensland, Australia

Corresponding author: SJ Stocks, GM PSTRC, suite 10, 7th Floor Williamson Building, University of Manchester, Oxford Road, Manchester, M13 9PL

Email jill.stocks@manchester.ac.uk

Tel: +44(0)1612751631 Fax: +44(0)1612757600

Funding statement: The National Institute for Health Research Greater Manchester Primary Care Patient Safety Translational Research Centre (NIHR GM PSTRC) and NHS Education for Scotland funded this study. The views expressed are those of the author(s) and not necessarily those of the NHS, the NIHR or the Department of Health.

Competing interests: None declared

**ABSTRACT**

**Background**

Never Events (NEs) are serious preventable patient safety incidents and are a component of formal quality and safety improvement (Q&SI) policies in the UK and elsewhere. A preliminary list of NEs for UK general practice has been developed but the frequency of these events, or their acceptability to GPs as a Q&SI approach, is currently unknown. The study aims to estimate i) the frequency of ten NEs occurring within GPs’ own practices; and ii) the extent to which the NE approach is perceived as acceptable for use.

**Methods** GPs were surveyed and mixed effects logistic regression models examined the relationship between: GP opinions of NE, estimates of NE frequency and the characteristics of the GPs and their practices.

**Results** Responses from 556 GPs in 412 practices were analysed. The majority of participants (70% to 88%, depending on the NE) agreed that the described incident should be designated a NE. Three NE were estimated to have occurred in less than 4% of practices in the last year, however, two NE were estimated to have occurred in 45% to 61% of practices. GPs reporting that a NE had occurred in their practice in the last year were significantly less likely to agree with the designation as a NE compared to GPs not reporting a NE (odds ratio 0.42; 95% CI 0.36, 0.49).

**Conclusions** The NE approach may have Q&SI potential for general practice but further work to adapt the concept and content is required.

**INTRODUCTION**

A substantial minority of patients unintentionally suffer harm as a result of their interactions with health care systems, including general practice.[1] Patient safety incidents in general practice are thought to be a relatively frequent occurrence, but most do not result in significant harm.[2] Many patient safety incidents may be preventable, creating a powerful rationale for initiatives to improve patient safety.[3] The World Health Organisation Safer Primary Care Expert Working Group recently recommended a systems approach towards reducing the occurrence of patient safety incidents.[4, 5]

Improving patient safety in general practice may require adapting approaches from safety-critical industries and other health care settings. There are many practical examples where this has been achieved, including: (i) the development and testing of a number of validated instruments to measure perceptions of safety culture;[6-8] (ii) application of the ‘Care Bundle’ approach to improve chronic disease management;[9] and (iii) application of the Trigger Review Method to patients’ medical records .[10]

A further example of a quality and safety improvement (Q&SI) initiative is the introduction of a ‘Never Events’ policy in 2009 to secondary care provided by the UK National Health Service (NHS).[11,12] A Never Event (NE) is a serious, largely preventable patient safety incident that should not occur if the available preventative measures were implemented by healthcare organisations.[13] They are relatively rare with 308 formal reports of NE occurring during 2014/15 in England.[14] Despite their apparent rarity, NE policies are considered worthwhile because of their potential benefits in terms of: (i) increasing awareness of priority patient safety issues; (ii) providing organisational support to pro-actively implement preventative measures; and (iii) formally acknowledging and dealing with serious patient safety incidents. NE could help to build a positive safety culture in general practice at the practice level by prioritising incidents for significant event analysis (SEA)[15] and/or be used to proactively review local safety systems. Formal reporting of NE could inform policy makers, researchers, educators, and frontline teams about the nature, scale and scope of patient safety incidents.

Ten NE specifically for general practice have been developed (Box 1, [16] but the frequency of these proposed NE is currently unknown. Given that around 90% of patient interaction is with NHS primary care services, it seems very likely that serious, preventable patient safety incidents do occur and some measure of the frequency would be desirable to inform patient safety initiatives in general practice.

This study aims to: (i) to determine the annual frequency of the proposed NE (Box 1) as estimated by UK general practitioners (GPs); (ii) to explore the extent to which the NE approach is acceptable to GPs; (iii) to examine the relationship between GP’s opinions and estimates as described in aims i and ii above and the characteristics of the GPs and their practices.

**METHODS**

**Questionnaire development and testing**

The survey was developed in an iterative manner. For each NE (Box 1) the GPs were asked Q1 to Q5 (Box 2) with the option to provide free text comments. Briefly, the questionnaire focused on respondents’ previous experiences of NE, their frequency of occurrence, estimates of risk of re-occurrence, perceptions of each incident as a NE, and actions taken in response to a NE. GPs were also asked to provide information about their practices and themselves. An initial version was piloted in Scotland by 15 GPs in December 2013; the final version survey was not substantially altered following the pilot.

**Setting and participants**

The questionnaire was administered concurrently to 519 general practices in Greater Manchester and all GP Educational Supervisors in Scotland (709 GPs within 332 practices representing approximately one quarter of all Scottish GPs) between 01 April 2014 and 31 May 2014.

**Data collection**

The questionnaire was completed online in Scotland and non-responders were followed-up on two occasions at 7-day intervals. In GM the questionnaire was distributed by post and non-responders were followed up on two occasions at 2-week intervals.

**Statistical methods**

The proportion of GPs or practices reporting NE to have occurred was reported. Estimates were collapsed into either categorical outcomes (frequency of NE = 0, 1, 2, 3 or more, Fig 1) or binary outcomes (NE did not occur or occurred at least once, Table 1). When asking about the frequency of NE occurrence during the last year (Q1, Box 2) only GPs that had worked for at least one year in their current practice were included (Table 1). For the practice estimates where there were disagreements in estimated frequencies between GPs the most frequently occurring estimate was used. If there were equal numbers of contradictory estimates of NE per practice (*i.e.* practices where 2 or 4 GPs had completed the questionnaire) both the lowest and highest estimates were reported (Table 1). In order to adjust the GP estimates for each NE for practice location (GM or Scotland) and allow for the clustering of GPs within practices a two-level logistic regression model was used (Tables 1 & 2). The practice location was included as a fixed effect and therefore adjusted the reported estimates whereas the practice identifier was included as a random effect and thereby provided more accurate confidence intervals (Tables 1&2). For the question asking about the level of agreement with the designation of the event as a NE (Q3, Box 2) the responses were dichotomised into a group expressing some level of agreement (“yes”, “probably” and “possibly”) and a group expressing definite disagreement (“no”, Table 2). The odds ratio that a GP would agree with the designation as a NE relative to NE1 (Prescribing aspirin for a patient ≤12 years, Box 1) was estimated using a standard one-level logistic regression model (Table 2).

To examine the relationship between the dichotomised responses to Q1 to Q4 and the characteristics of the GPs and their practices across all the NE, four versions of a three-level logistic regression model were fitted (Table 3). The three-level model allows for different outcomes for each NE and takes clustering of GPs within practices in to account by including categorical variables identifying each NE and practice as random effects in the model.

Model 1 – associations between reporting a NE (Q1&2, Box 2) and GP/ practice characteristics

Model 1 examined the associations between a binary outcome variable indicating whether or not a NE occurred at least once, or did not occur, and the following predictors; years worked as a GP, partner or salaried GP, gender, part or full-time working pattern, practice list size, location (Scotland or GM) and whether the practice was an accredited training practice (all Scottish practices were training practices). The odds ratios from the regression model give a relative measure of how likely GPs were to estimate that a NE had occurred in their practice according to the characteristics listed above.

Model 2 – associations between agreement with the designation as a NE (Q3, Box 2), the reporting of a NE in past year (Q1, Box 2) and GP/ practice characteristics

Model 2 examined the associations between a binary outcome variable indicating whether or not a GP agreed with the designation of the event as a NE (answered “yes”, “probably” and “possibly” to Q3) or disagreed (answered “no” to Q3). The predictors were the same as in model 1 (listed above) plus a variable indicating whether or not the GP had estimated a NE to have occurred in the last year. The odds ratio for this additional variable estimates the impact of working in a practice where a NE had occurred in the last year on the GP’s opinion about agreement or disagreement with the designation of the event as a NE (Q3, Table 2).

Model 3 – associations between estimation that NE will occur within the next 5 years (Q4 Box 2), the reporting of a NE in past year (Q1, Box 2) and GP/ practice characteristics

Model 3 examined the associations between a binary outcome variable indicating GP’s opinion about the likelihood that a NE would occur in the next 5 years. The responses to Q4 were dichotomised in to groups estimating that it was very unlikely that a NE would occur in the next 5 years (no chance or very unlikely to happen) versus those estimating it could happen (unlikely through certain to happen). The predictors were the same as in model 2 (listed above).

Model 4 – associations between providing free text comments, the designation as a NE (Q3, Box 2), the reporting of a NE in past year (Q1, Box 2) and GP/ practice characteristics

Model 4 examined associations between providing free text comments, or not, and used same predictors as model 2 (listed above) plus a variable indicating the GPs level of agreement with the statement that this was a NE (Q3). The odds ratios for the predictors help to describe the characteristics and opinions of the GPs who provided free text comments.

**Content analysis**

A bottom-up (inductive) approach was used to identify similar topics within the comments. One author (SJS) read the comments in a random order *i.e.* not within their NE grouping and identified the most frequent topics. Each comment was coded accordingly and as new topics emerged the comments were recoded and similar codes were merged. The process ended when each comment had at least one code although some comments had multiple codes when several topics were addressed in the same comment. The coding was checked by a different author (RA) and any disagreements were resolved by discussion.

**RESULTS**

**Response rates**

In Scotland 283 GPs representing 215 practices responded to the questionnaire (practice response rate=65%; GP response rate=40%). Two GPs were excluded from the analysis (1 retired and 1 locum GP). In GM 282 GPs representing 202 practices responded (practice response rate=39%; GP response rate not available as invitations were at the practice level). Seven GM physicians were excluded from the analysis (2 did not work in general practice, 3 were other types of clinician working in general practice, 1 locum GP and 1 GP only completed one question). Following these exclusions questionnaires from 281 GPs in 214 practices in Scotland and 275 GPs in 198 practices in GM were analysed (412 practices in total). More than one GP completed the questionnaire in 109/412 (26%) practices. In 96/412 (23%) practices 2 GPs completed the questionnaire, in 12/412 (3%) 3 GPs and 1/412 (<1%) 4 GPs.

**Professional characteristics and practice demographics**

All Scottish GPs were educational trainers therefore their practices were designated as training practices. In GM 48% of practices were training practices (91/191, 7 not known). Across both locations the majority of GPs were partners rather than salaried (90%; 499/555, 1 missing), 56% were working part-time (312/555, 1 missing) and there were equal numbers of each gender (51% male 284/554, 2 missing). GPs tended to be experienced, particularly the Scottish GPs (mean time worked as a GP; Scotland =19.1 years; sd ±7.4 years, GM =17.2 years; sd ±9.4 years, P<0.001). There were no differences in mean list size between Scotland (7482; ±2997) and GM (7481; ±3848, P=0.997) but Scottish practices had more partners (5.3; ±2.0) compared with GM (3.9; ±2.0, P<0.001).

**Estimated frequency of NE**

The estimated frequency of occurrence for each NE during the last year is shown in Figure 1 (upper estimates) and Table 1. Four practices were excluded because the sole GP responding had been in post for less than 1 year. Furthermore, due to an administrative error, 45/198 (23%) of the GM practices and 48/275 (17%) GM GPs were not asked to estimate the frequency of never events during the last year (Q1) but the full response set was available for all other questions.

The proportion of practices estimating that a NE had occurred in the past year ranged from <1% to 61% (comparing lower estimate for prescribing aspirin for a patient <12 years with upper estimate for abnormal investigation result not reviewed, Table 1). Three of the 10 never events were estimated to have occurred in the last year in 4% or fewer practices (“Prescribing aspirin for a patient <12”, “Methotrexate daily rather than weekly”, “Adrenaline is not available when needed”, Table 1). Conversely two NE were estimated to have occurred in the last year in 45% to 61% of practices (“Abnormal investigation result not reviewed”, “Prescribing when adverse reaction recorded”, Table 1).

**Estimated likelihood that NE will occur in future, level of agreement with the designation as a NE and actions following a NE**

The proportion of GPs who estimated that NE were definitely or likely to happen in the next 5 years ranged from 8% (Prescribing aspirin for a patient <12 years) to 63% (Abnormal investigation result not reviewed, Table 1). A more detailed breakdown of the response to this question is shown in Table C, online appendix 1, http://links.lww.com/JPS/A85. The proportion of GPs responding “no” to Q3 (Do you think this incident is a 'Never Event'?) ranged from 12% to 30% (Table 2). Of the 556 GPs 551 (99%) reported they would undertake a SEA in response to a NE and 141 (25%) would also submit a formal incident report. Of the 5 GPs remaining 2 (<1%) would discuss the NE at a practice meeting and 3 (<1%) did not report any actions but other GPs in the same practice reported they would take one of the actions listed above.

**Results of the multilevel regression models**

The relative importance of the patient and practice characteristics (predictors) in determining the outcome (response to Q1 to 4 etc) are shown as odds ratios generated by the regression models in Table 3.If the 95% confidence intervals for an odds ratio do not overlap the reference category (*i.e.* 1) it is considered to be significantly different to the reference category (and highlighted in bold). The P value for an odds ratio significantly different to the reference category is therefore less than 0.05 (but could be smaller). The odds of estimating a never event had occurred in the last year (Q1) were doubled in practices with a list size over 12000 relative to those under 5000. Scottish GPs and less experienced GPs (under 5 years work experience) were significantly more likely to report a NE (model 1, Table 3).

GPs who had estimated that a NE had occurred in the last year were significantly less likely to agree it should be designated as a NE but female GPs, part-time GPs, Scottish GPs, partners rather than salaried GPs and GPs working in training practices were all significantly more likely to agree that the event should be described as a NE (model 2, Table 3).

The strongest predictor of a GP being of the opinion that a NE would occur in the next 5 years (Q4) was the frequency of past occurrences. Less experienced, salaried and Scottish GPs were significantly more likely to estimate that a NE would occur in the next 5 years (model 3, Table 3).

GPs that were undecided about the designation as a NE were most likely to provide free text comments (GPs answering “probably” to the Q4 is it a NE were 15 times more likely to provide a comment than those answering “no” and 30 times more likely than those answering “yes”). GPs who answered that a NE had occurred in their practice, GPs working in a training practice and more experienced GPs were more likely to comment and Scottish GPs were less likely to comment than GM GPs (model 4, Table 3).

**Results of content analysis of free text comments**

In total 1025 comments were provided, most made one main point (72%) but some addressed multiple points (28%). The comments tended to be very specific to an individual NE and altogether they fell into 28 groups making a substantively similar point (Table 4). Some comments suggested changes to the NE to make it more acceptable (Table A, online appendix 1, http://links.lww.com/JPS/A85). A detailed summary of the categories of comments for each NE is shown in Tables B1 to B10 (online appendix 1, http://links.lww.com/JPS/A85).

**DISCUSSION**

**Summary of Results**

The study findings suggest that the designation of a NE as a NE is dependent on the individual/type of NE and that on the whole, NEs were reportedly rare. Although GPs were more likely to disagree with the NE label for the more frequently occurring NEs, this was not in proportion to their increased frequency of occurrence. For example a “cancer referral not sent” was around 10 times more likely to have occurred than “Methotrexate prescribed daily rather than weekly” (Table 1) but GPs were only about 1.5 times more likely to disagree with the designation as a NE (Table 2). Most GPs, however, remained unconvinced that the risk can be eliminated for any of the NEs (Table 1, Table C in online appendix 1, http://links.lww.com/JPS/A85). GPs do however appear to take the actual and potential occurrence of such events seriously given that 99% stated an intention to undertake a SEA [17] following a NE. Free-text comments originated mainly from GPs who were undecided about the labelling of the event as a NE. Opinions varied widely with some GPs commenting that the risk of serious harm was extremely low whereas other GPs suggested that the NE should be more stringent. Some GPs felt that the NE description was placing a burden of responsibility on them that was not intended by the description of the NE, *e.g.* that they should be responsible for the actions of a laboratory or the ambulance service. There were differences in opinion about the level of responsibility a GP should take for the actions of non-medical staff.

**Strengths, limitations and generalisability of the study**

This is the first study to attempt to estimate the frequency of a preliminary set of NEs for UK general practice and as such will help to inform whether or not an NE policy should be rolled out across UK general practice. Despite its potential contribution, the study does have some limitations. Firstly, unfortunately, some GM GPs were not asked the first question due to a mistake when printing the questionnaires and these practices were excluded from the analysis of this question. Secondly, included practices may not be generalizable to all of the UK; participating practices tended to be larger than their respective national averages (list size 7482 vs 5622 in Scotland [18] & 7481 vs 6487 in England.[19] Furthermore the sample is self-selected and includes only GP trainers in Scotland. In GM, training practices were more likely to respond to the survey (48% compared to around 40% of all practices in Central and South Manchester). This might be a source of bias given that training practices were more likely to agree with the description as a NE, were more optimistic about the likelihood of NE happening in future and more likely to make a free text comment (Table 3). Thirdly, we must emphasize that these results and analyses are based on retrospective and subjective estimates by GPs and we do not know how often NEs might truly occur in primary care. Furthermore, certain events may more easily be recalled than others and may also bias the reported frequencies. The estimates are intended to inform the reader about the potential usefulness of the Never Event approach in demonstrating GPs recognise these events and state that they do happen, rather than estimate the *actual* frequency of occurrence.

**Implications for research and practice**

Our results show that the NE approach does not transfer easily from secondary care to primary care. Nonetheless, NEs may have a role in general practice with some refinement of the content and purpose. For example, NEs 1-5 (Box 1) are predominantly errors of commission; they rarely occur and GPs are more likely to agree with their designation as a NE. They are too rare to be used to identify practices in need of intervention but could be used at a system level to draw attention to patient safety. Focussing on rare events at the practice level may not be the best use of limited resources, indeed it seems counterintuitive.[19] The more frequently reported NEs (6-10, Box 1) are predominantly errors of omission and GPs are less likely to agree with their designation as a NE. In general practice, however, mild to moderate harm is more commonly associated with errors of commission and severe harm is more commonly associated with errors of omission such as misdiagnosis or delayed diagnosis.[5] This contrasts with secondary care where NE are predominantly errors of commission. So although NE (6-10) are more likely to be associated with more serious harm and occur more frequently, they are less likely to be recognised as NE by GPs. NE 6-10 could be expanded in to a set of markers for Q&SI, possibly by going back to the original 50 NE from which this list of 10 was developed. [16] Selected subsets could then be adapted locally to suit individual contexts and preferences and weighting of particular NE might help to make them acceptable to GPs. It is also difficult to compare the rates estimated here with those reported in secondary care as the denominators differ *e.g.* 340 million general practice consultations annually compared with 19 million episodes for patients admitted to hospital. [20, 21] A crude scaling up (assuming 8000 practices in England) implies around 50 occurrences of “Prescribing aspirin for a patient ≤12 years” and 300 of “Methotrexate prescribed daily rather than weekly” per year compared to around 300 NE reported in English hospitals in 2014. [14] Secondly the potential to cause serious harm following a NE in general practice is less clear cut than in secondary care; there may the opportunity to remove or mitigate the consequences of the NE in the future and all the definitions include caveats and exceptions.

In our study, the content analysis revealed that the locus of responsibility for the occurrence of NEs was also an issue. In secondary care, the lines of responsibility and boundaries of operations may be more easily defined as a closed system, whereas general practice is more diffuse and potentially viewed in comparison to secondary care, as an open system. [22] Other work for example has pointed out the importance of receptionists in reducing the likelihood of a medical error. [23]

Finally**,** given that GPs are less likely to agree with the NE designation if they reported a NE in their practice, it seems possible that they might view the NE as a negative comment on their practise and would need to be reassured that the aim of a NE policy is to identify weaknesses in the system and *prevent* NEs from occurring, rather than a way to punitively judge general practice. Ostensibly, replacing the “Never Events” label with one that better reflects the missed opportunities to improve patient safety might improve their acceptability to GPs.

**Conclusion**

In a resource-limited and over-stretched system there may be a trade-off when addressing comprehensive patient safety. We suggest that further work could explore expanding the list of more frequently-occurring NE (*e.g.* 6-10) as a Q&SI approach at a practice level and the rarer NE (1-5) could be useful for surveillance at a system level and to draw attention to broader safety issues. The “Never Events” label could be replaced with one that better reflects the missed opportunities for NE 6-10 *e.g.* “Serious Preventable Events” but could be retained for NE 1-5.

**ACKNOWLEDGMENTS**

Thank you to all the GPs who completed the survey and Dr Julie Ferguson for administering the survey in Scotland.

**ETHICAL APPROVAL**: University of Manchester Ethics Committee 2 Approval 14027

**DATA SHARING STATEMENT**: Raw data (numerical only) is available from jill.stocks@manchester.ac.uk

**REFERENCES**

1. Francis R. Report of the Mid Staffordshire NHS Foundation Trust Public Inquiry. Executive summary 2013. <http://www.midstaffspublicinquiry.com/report> (accessed 2 Jan2016).

2. Panesar SS, deSilva D, Carson-Stevens A, et al. How safe is primary care? A systematic review. *BMJ Qual Saf 2015*; Published Online First doi:10.1136/bmjqs-2015-004178 <http://dx.doi.org/10.1136/bmjqs-2015-004178> (accessed 10 May 2016).

3. Tsang C, Majeed A, Aylin P. Routinely recorded patient safety events in primary care: a literature review. *Fam Pract* 2012;29:8-15.

4. Cresswell KM, Panesar SS, Salvilla SA, et al. Global research priorities to better understand the burden of iatrogenic harm in primary care: an international Delphi exercise. *PLoS medicine* 2013;10:e1001554

5. The WHO Safer Primary Care Expert Working Group. Safer Primary Care. A Global Challenge. Summary of Inaugral Meeting 2012. WHO Press, World Health Organization, Geneva <http://www.who.int/patientsafety/summary_report_of_primary_care_consultation.pdf> (accessed 10 May 2016)

6. Kirk S, Parker D, Claridge T, et al. Patient safety culture in primary care: developing a theoretical framework for practical use. *Qual Saf Health Care* 2007;16:313-20.

7. de Wet C, Spence W, Mash R, et al. The development and psychometric evaluation of a safety climate measure for primary care *Qual Saf Health Care* 2010;19:578-84.

8. Newham R, Bennie M, Maxwell D, et al. Development and psychometric testing of an instrument to measure safety climate perceptions in community pharmacy. *J Eval Clin Pract* 2014;20:1144-52.

9. de Wet C, McKay J, Bowie P. Combining QOF data with the care bundle approach may provide a more meaningful measure of quality in general practice. *BMC Health Serv Res* 2012;12:351. <http://www.biomedcentral.com/1472-6963/12/351> (accessed 2 Jan 2016).

10. Bowie P, Halley L, Gillies J, et al. Searching primary care records for predefined triggers may expose latent risks and adverse events. *Clin Risk* 2012;18:13-8.

11. Never Event – Framework: Process and action for Primary Care Trusts 2009-10. National patient Safety Agency 2009. <http://www.nrls.npsa.nhs.uk/neverevents/?entryid45=59859> (accessed 2 Jan 2016).

12. Never Events List 2015/16. NHS England Patient Safety Domain 2015. <https://www.england.nhs.uk/patientsafety/wp-content/uploads/sites/32/2015/03/never-evnts-list-15-162.pdf> (accessed 2 Jan 2016).

13. Revised Never Events Policy and Framework. NHS England Patient Safety Domain 2015. <https://www.england.nhs.uk/patientsafety/wp-content/uploads/sites/32/2015/04/never-evnts-pol-framwrk-apr2.pdf> (accessed 2 Jan 2016).

14. Provisional publication of Never Events reported as occurring between 1 April 2014 and 31 March 2015. NHS England Patient Safety Domain 2015. <https://www.england.nhs.uk/wp-content/uploads/2015/07/provsnl-ne-data-04-14-to-03-15.pdf> (accessed 2 Jan 2016).

15. Bowie P ME, Bruce D. Enhancing the effectiveness of significant event analysis: exploring personal impact and applying systems thinking in primary care. *Journal of Continuing Education in the Health Professions* 2016; In press.

16. de Wet C, O'Donnell C, Bowie P. Developing a preliminary 'never event' list for general practice using consensus-building methods. *Br J Gen Pract* 2014;64:e159-67.

17. Bowie P, de Wet C, Pringle M. Significant Event Analysis Guidance for Primary Care teams. NHS Education for Scotland 2011. <http://www.nes.scot.nhs.uk/media/346578/sea_-_full_guide_-_2011.pdf> (accessed 2 Jan 2016).

18. General Practice – GP Workforce and Practice Population Statistics 2013. NHS Scotland 2013. <https://isdscotland.scot.nhs.uk/Health-Topics/General-Practice/Publications/2013-12-17/2013-12-17-GPWorkforce2013-Summary.pdf?71755617857> (accessed 2 Jan 2016).

19. Runciman WB, Edmonds MJ, Pradhan M. Setting priorities for patient safety. *Quality & safety in health care* 2002;11:224-9.

20. Gregory S. General practice in England: An overview. The King’s Fund 2009. <http://www.kingsfund.org.uk/sites/files/kf/general-practice-in-england-overview-sarah-gregory-kings-fund-september-2009.pdf> (accessed 2 Jan 2016).

21. Hospital Episode Statistics Admitted Patient Care, England-2014-15. Health and Social Care Information Centre 2015. <http://www.hscic.gov.uk/catalogue/PUB19124/hosp-epis-stat-admi-summ-rep-2014-15-rep.pdf> (accessed 2 Jan 2016).

22. Von Bertalanffy L. The History and Status of General Systems Theory The Academy of Management Journal 1972;15:407-26 http://www.jstor.org/stable/255139 (accessed 22 Feb 2017)

23. Litchfield I, Bentham L, Hill A, et al. Routine failures in the process for blood testing and the communication of results to patients in primary care in the UK: a qualitative exploration of patient and provider perspectives. *BMJ Qual Saf* 2015;24:681-90.

**FIGURE LEGEND**

Fig 1.Frequency of “never events” occurring in the past 12 months as estimated by GPs (% practices with zero never events). The higher estimate is shown in cases of disagreement between GPs within the same practice.

Box 1. List of Never Event labels and description [16]

**1. Prescribing aspirin for a patient ≤12 years**

- Prescribing Aspirin for a patient ≤ 12 years old (unless recommended by a specialist for specific clinical conditions e.g. Kawasaki’s disease)

**2. Methotrexate prescribed daily rather than weekly**

- Prescribing Methotrexate daily rather than weekly (unless initiated by a specialist for a specific clinical condition e.g. leukemia)

**3. Adrenaline is NOT available when needed**

- Adrenaline/Epinephrine is NOT available within minutes when clinically indicated for a medical emergency in the practice or GP home visit

**4. Prescribed teratogenic drug when pregnant**

- Prescribing a teratogenic drug to a patient the clinician knows to be pregnant (unless advised to do so by a clinical specialist)

**5. Prescribed HRT & has intact uterus**

- Prescribing systemic oestrogen-only Hormone Replacement Therapy for a patient with an intact uterus

**6. Cancer referral not sent**

- A planned referral of a patient, prompted by clinical suspicion of cancer, is not sent

**7. Ambulance transport is not arranged**

- Ambulance transport is not arranged if this had been agreed when deciding to admit a patient as an emergency

**8. Needle stick injury due to sharps disposal failure**

- A needle-stick injury due to a failure to dispose of ‘sharps’ in compliance with national guidance and regulations

**9. Prescribing when adverse reaction recorded**

- Prescribing a drug to a patient that has correctly been recorded in the practice system as having previously caused her/him a severe adverse reaction

**10. Abnormal investigation result is not reviewed**

- An abnormal investigation result is received by a practice but is not reviewed by a clinician

Box 2. Questions asked for each Never Event (Q1-Q4) and for all Never Event (Q5)

Q1. Based on your experience, please indicate the number of times this event has occurred in your current practice in the last year?

Q2. Based on your experience, please indicate the number of times this event has occurred in your current practice or any other practice you have ever worked?

Q3. Do you think this incident is a 'Never Event'?

Q4. Please estimate the risk of this event occurring in your practice in the next 5 years

Q5. If you become aware in the future that any of the previous incidents have occurred in your practice, which of the following actions would you be prepared to undertake?

- Informal discussion with colleague(s) involved in the incident
- A significant event analysis (SEA)
- Submit a formal incident report to a local health authority or other organisation
- Discuss the incident at a practice meeting
- Adopt a 'watch and see' approach if no patient harm occurred

Table 1. GP estimates of the frequency of Never Events occurring in the last year and in future

| NE – short description | Has a NE occurred in your current practice in the last year?(Q1, Box 2) | | | GP estimates that NE will occur in next 5 years^4^  (Q4, Box 2)  n=556  (missing) |
| --- | --- | --- | --- | --- |
|  | Proportion of GPs estimating  occurred at least once  n=501^1^ (missing) | Adjusted proportion of GPs estimating  occurred at least once^2^ (95% CI) | Proportion of practices estimating occurred at least once  n=382 (missing) |  |
| Prescribing aspirin for a patient ≤12 years | 6 (1) 1% | 1%  (0 to 4%) | 2 to 6 (0)  <1% to 2%^3^ | 41 (11) 8% |
| Methotrexate prescribed daily rather than weekly | 17 (1) 3% | 4%  (2 to 7%) | 13 to 16 (0)  3% to 4% | 51 (5) 9% |
| Adrenaline is NOT available when needed | 20 (0) 4% | 7%  (5 to 13%) | 12 to 17 (1)  3% to 4% | 127 (7) 23% |
| Prescribed teratogenic drug when pregnant | 37 (1) 7% | 8%  (5 to 13%) | 19 to 36 (0)  5% to 9% | 159 (11) 29% |
| Prescribed HRT & has intact uterus | 73 (1) 15% | 11%  (8 to 16%) | 46 to 66 (2)  12% to 17% | 202 (5) 37% |
| Cancer referral not sent | 148 (1) 30% | 27%  (20 to 36%) | 105 to135 (1)  28% to 35% | 198 (6) 36% |
| Ambulance transport is not arranged | 95 (2) 19% | 8%  (3% to 18%) | 66 to 85 (2)  17% to 22% | 160 (9) 29% |
| Needle stick injury due to sharps disposal failure | 108 (1) 22% | 10%  (5% to 19%) | 74 to 99 (0)  19% to 26% | 271 (3) 49% |
| Prescribing when adverse reaction recorded | 258 (2) 52% | 41%  (34% to 48%) | 170 to 218 (0)  45% to 57% | 335 (3) 61% |
| Abnormal investigation result is not reviewed | 277 (3) 56% | 44%  (37% to 52%) | 187 to 231 (1)  49% to 61% | 346 (4) 63% |

^1^excludes GPs working for less than one year in their current practice

^2^proportion predicted by a 2-level regression model adjusted for location (GM or Scotland) and clustering within practices (see methods)

^3^lower and upper estimates reflect minimum and maximum estimated frequencies for practices with disagreement between GPs within the same practice (see methods)

^4^ proportion responding “unlikely”, “moderate chance”, “likely”, “very likely” or “certain to happen” to Q3. For more detail see Table C in online appendix 1, http://links.lww.com/JPS/A85.

Table 2. The number of GPs disagreeing with the designation as a Never Event (answered “no” to Q3, Box 2)

| NE – short description | Do you think this incident is a 'Never Event? (Q3, Box 2) | | |
| --- | --- | --- | --- |
|  | Proportion of GPs responding “no” (%)  n=556 | Adjusted proportion of GPs responding “no” ^1^ (95% CI) | Odds ratio^2^  (relative likelihood will answer “no”)  (95% CI) |
| Prescribing aspirin for a patient ≤12 years | 87 (16%) | 19%  (15% to 24%) | 1 (reference) |
| Methotrexate prescribed daily rather than weekly | 65 (12%) | 13%  (10% to 18%) | 0.71  (0.51 to 1.01) |
| Adrenaline is NOT available when needed | 83 (15%) | 18%  (14% to 23%) | 0.95  (0.68 to 1.31) |
| Prescribed teratogenic drug when pregnant | 79 (14%) | 15%  (11% to 19%) | 0.89  (0.64 to 1.24) |
| Prescribed HRT & has intact uterus | 111 (20%) | 20%  (16% to 26%) | 1.34  (0.99 to 1.83) |
| Cancer referral not sent | 98 (18%) | 17%  (13% to 22%) | 1.15  (0.84 to 1.58) |
| Ambulance transport is not arranged | 133 (24%) | 21%  (17% to 26%) | 1.70  (1.26 to 2.29) |
| Needle stick injury due to sharps disposal failure | 164 (30%) | 23%  (18% to 28%) | 2.26  (1.68 to 3.02) |
| Prescribing when adverse reaction recorded | 118 (21%) | 20%  (16% to 25%) | 1.45  (1.07 to 1.97) |
| Abnormal investigation result is not reviewed | 135 (24%) | 22%  (17% to 27%) | 1.73  (1.28 to 2.33) |

^1^proportion predicted by a 2-level regression model adjusted for location (GM or Scotland) and clustering within practices (see methods)

^2^odds ratio relative to the NE “Prescribing aspirin for a patient ≤12 years” derived from a one-level logistic regression model using responses to Q3 (GPs answering no)/(GPs answering yes, probably, possibly)

Table 3. Adjusted odds ratios for GP and practice level predictors from a 3 level logistic regression model with random effects at the Never Event and practice level

| Predictors | Q1.NE happened in last year^1^  (model 1)^2^ | Q2.NE happened ever in working life (model 1)^2^ | Q3. Agree with designation as a never event? (model 2)^3^ | Q4. Estimate NE will happen in future (model 3)^4^ | Made a comment about at least one NE (model 4)^5^ |
| --- | --- | --- | --- | --- | --- |
| List size |  |  |  |  |  |
| *≤5000* | 1 (reference) | 1 (reference) | 1 (reference) | 1 (reference) | 1 (reference) |
| *5001 – 8000* | 1.13 [0.89, 1.43] | **1.30 [1.08, 1.57]** | 0.97 [0.82, 1.16] | 1.06 [0.88, 1.28] | 0.75 [0.60, 0.94] |
| *8001 – 12000* | 1.20 [0.94, 1.52] | **1.55 [1.29, 1.88]** | 0.92 [0.77, 1.09] | 1.04 [0.86, 1.26] | 0.78 [0.62, 0.97] |
| *12001 – 22000* | **2.06 [1.50, 2.83]^6^** | **2.29 [1.76, 2.98]** | 0.98 [0.77, 1.23] | 1.02 [0.79, 1.32] | 0.67 [0.49, 0.92] |
| Years as a GP |  |  |  |  |  |
| *<=5* | 1 (reference) | 1 (reference) | 1 (reference) | 1 (reference) | 1 (reference) |
| *>5-10* | **0.58 [0.39, 0.87]** | 0.97 [0.70, 1.33] | 0.99 [0.75, 1.31] | **0.61 [0.45, 0.83]** | **1.55 [1.05, 2.30]** |
| *>10-20* | 0.73 [0.51, 1.05] | 1.31 [0.97, 1.75] | 1.01 [0.79, 1.30] | **0.54 [0.41, 0.71]** | 1.41 [0.98, 2.02] |
| *>20-44* | **0.57 [0.39, 0.82]** | 1.17 [0.87, 1.56] | 1.20 [0.93, 1.54] | **0.36 [0.27, 0.48]** | **1.54 [1.07, 2.12]** |
| Location |  |  |  |  |  |
| *Manchester* | 1 (reference) | 1 (reference) | 1 (reference) | 1 (reference) | 1 (reference) |
| *Scotland* | **2.11 [1.65, 2.69]** | **2.22 [1.84, 2.68]** | **1.21 [1.03, 1.43]** | **1.53 [1.27, 1.84]** | **0.67 [0.54, 0.83]** |
| Position |  |  |  |  |  |
| *partner* | 1 (reference) | 1 (reference) | 1 (reference) | 1 (reference) | 1 (reference) |
| *salaried* | 1.24 [0.88, 1.74] | 1.07 [0.83, 1.39] | **0.68 [0.54, 0.85]** | **1.84 [1.43, 2.37]** | 1.21 [0.88, 1.65] |
| Gender |  |  |  |  |  |
| *male* | 1 (reference) | 1 (reference) | 1 (reference) | 1 (reference) | 1 (reference) |
| *female* | 1.09 [0.89, 1.33] | 1.05 [0.90, 1.23] | **1.35 [1.17, 1.56]** | 1.01 [0.87, 1.18] | 1.04 [0.86, 1.26] |
| Work pattern |  |  |  |  |  |
| *full-time* | 1 (reference) | 1 (reference) | 1 (reference) | 1 (reference) | 1 (reference) |
| *part-time* | 0.84 [0.69, 1.02] | 1.0 [0.85, 1.17] | **1.20 [1.04, 1.38]** | 0.86 [0.73, 1.00] | 0.88 [0.73, 1.06] |
| Training status |  |  |  |  |  |
| *not training practice* | 1 (reference) | 1 (reference) | 1 (reference) | 1 (reference) | 1 (reference) |
| *training practice* | 0.81 [0.61, 1.08] | 1.16 [0.94, 1.43] | **1.31 [1.09, 1.58]** | **0.77 [0.62, 0.95]** | **1.43 [1.11, 1.84]** |
| Ever reported NE |  |  |  |  |  |
| *not reported NE* | - | - | 1 (reference) | 1 (reference) | 1 (reference) |
| *reported NE* | - | - | **0.42 [0.36, 0.49]** | **4.94 [4.09, 5.97]** | **1.48 [1.19, 1.84]** |
| Is it a NE? |  |  |  |  |  |
| *Not a NE* | - | - | - | - | 1 (reference) |
| *Possibly* | - | - | - | - | **3.48 [2.49, 4.87]** |
| *Probably* | - | - | - | - | **15.25 [9.97, 23.32]** |
| *Yes* | - | - | - | - | 0.56 [0.46, 0.69] |

^1^excludes GPs working less than 1 year in current practice; ^2^odds ratio gives a relative measure of likelihood that a GP within the group defined by the predictor reported that a NE has occurred in their practice or ^3^agreed that the event should be designated a NE (answered yes, probably or possibly to Q3 Box 2) or ^4^estimated that a NE is certain or very likely to happen in the next five years or ^5^provided explanatory free text responses; bold text indicates where 95% confidence intervals do not include 1 and are considered to be significantly different to the reference category (1)

^1^P(χ 2) = probability there is no difference between English and Scottish GPs in estimated number of never events

| Common topics raised in GP comments  Table 4. Counts of themes identified within the comments provided by GPs | Count of comments relevant to each topic (%) | | | | | | | | | | |
| --- | --- | --- | --- | --- | --- | --- | --- | --- | --- | --- | --- |
|  | NE 1  Aspirin & u16  n=70 | NE 2  Mtx daily  n=93 | NE 3  Arenal-ine n/a  n=188 | NE 4  Terato-genic  n=50 | NE 5  HRT & uterus  n=93 | NE 6 Cancer referral  n=125 | NE 7 Ambul-ance  n=136 | NE 8 Needle stick  n=95 | NE 9  ADR  n=197 | NE 10  No review  n=299 | All NE  n=1367 |
| Suggested changes or clarification | 13  (19%) | 7  (8%) | 20  (11%) | 9  (18%) | 15  (16%) | 9  (7%) | 25  (18%) | 21  (22%) | 18  (19%) | 49  (16%) | 186  (14%) |
| Description of prevention strategies or sharing reasons behind the NE | - | 14  (15%) | 37  (20%) | 2  (4%) | 15  (16%) | 21  (17%) | 23  (17%) | 12  (13%) | 6  (3%) | 20  (7%) | 171  (13%) |
| Computerised systems are helpful to prevent NE | 12  (17%) | 45  (48%) | - | - | 8  (9%) | 5  (4%) | - | - | 23  (12%) | 22  (7%) | 115  (8%) |
| “Human error” is inevitable or NE is difficult to prevent | - | - | - | - | 10  (11%) | 29  (23%) | 22  (16%) | 29  (31%) | 9  (5%) | 12  (4%) | 111  (8%) |
| Home visits increase the risk of the NE happening | - | - | 60  (32%) | - | - | - | - | 13  (18%) | 29  (15%) | - | 102  (7%) |
| Computerised systems are unhelpful or increase risk of NE | - | 6  (6%) | - | - | 3  (3%) | 13  (10%) | - | - | 29  (15%) | 13  (4%) | 64  (5%) |
| NE description needs to be more specific *e.g.* the level of the teratogenicity or severity of adverse drug reaction | - | - | - | 14  (28%) | - | - | - | - | - | 21  (7%) | 35  (3%) |
| NE can occur due to error outside of general practice *e.g.* ambulance, laboratory | - | 5  (5%) | - | - | 5  (5%) | 6  (5%) | 28  (21%) |  | 2  (1%) | 7  (2%) | 53  (4%) |
| NE so rare it is not a useful measure of quality or is a historical problem | 17  (24%) |  | 25  (13%) | - | 5  (5%) | - | - | - | - | - | 47  (3%) |
| NE occurs due to large volume of results or referrals, insufficient time & work load, GP absence | - | - | - | - | - | 7  (6%) | - | - | - | 26  (9%) | 33  (2%) |
| NE can occur due to incorrect or poor record keeping | - | - | - | - | 3  (3%) | - | - | - | 30  (19%) | - | 33  (2%) |
| More likely that abnormal result missed or inappropriate action taken | - | - | - | - | - | - | - | - | - | 34  (11%) | 34  (2%) |
| NE can occur for valid clinical reasons | 5  (7%) | - | - | 12  (24%) | 12  (13%) | - | - | - | 29  (15%) | - | 58  (4%) |
| Administrative errors can play a major role including poor communication | - | - | - | - | - | 5  (4%) | 12  (9%) | - | - | 17  (6%) | 34  (2%) |
| Delay is more likely than referral not sent or result not reviewed | - | - | - | - | - | 13  (10%) | - | - | - | 22  (7%) | 35  (3%) |
| Specific to an individual *e.g.* trainee or problem individual | 5  (7%) | - | - | - | 10  (11%) | 3  (2%) | - | 8  (8%) | 3  (2%) | - | 29  (2%) |
| Patients can increase the risk of a NE *e.g.* not say are pregnant |  | - | - | - | 4  (4%) | 2  (2%) | 7  (5%) | - | 2  (1%) | 2  (1%) | 17  (1%) |
| Patients can be helpful in preventing NE | - | - | - | 9  (18%) | - | 9  (7%) | 9  (7%) | - | 3  (2%) | 3  (1%) | 33  (2%) |
| Medicine out of date more likely than not available | - | - | 23  (12%) | - | - | - | - | - | - | - | 23  (2%) |
| Not all abnormal results are clinically significant | - | - | - | - | - | - | - | - | - | 25  (8%) | 25  (2%) |
| Unclear who is responsible for checking the result | - | - | - | - | - | - | - | - | - | 17  (6%) | 17  (1%) |
| There may be a better option to deal with the situation *e.g.* call ambulance | - | - | 14  (7%) | - | - | - | - | - | - | - | 14  (1%) |
| Risk of harm to patient due to NE is very small | 9  (13%) | - | - | - | - | - | - | - | - | - | 9  (<1%) |
| Pharmacists can play a role in preventing NE | - | 6  (6%) | - | - | 1  (1%) | - | - | - | 2  (1%) | - | 9  (<1%) |
| By definition the GP may be unaware of the NE | - | - | - | - | - | - | - | - | - | 9  (3%) | 9  (<1%) |
| Danger is more to another individual than patient *e.g.* cleaner | - | - | - | - | - | - | - | 7  (7%) | - | - | 7  (<1%) |
| Other | 9  (13%) | 10  (11%) | 9  (5%) | 4  (8%) | 2  (2%) | 3  (2%) | 10  (7%) | 5  (5%) | 12  (6%) | 0  (0%) | 64  (5%) |
